# T244. SELF-DEFINING MEMORIES PREDICT ENGAGEMENT IN STRUCTURED ACTIVITY IN FIRST EPISODE PSYCHOSIS

**DOI:** 10.1093/schbul/sby016.520

**Published:** 2018-04-01

**Authors:** Abigail Wright, Geoff Davies, David Fowler, Kathryn Greenwood

**Affiliations:** University of Sussex

## Abstract

**Background:**

Self-defining memories (SDM) are vivid personal events, related to important life memories and narrative identity. Self-defining memories reported by individuals with schizophrenia have been found to be less specific, more negative, and individuals extract less meaning from the memories compared to a healthy control group. Research in healthy control participants has demonstrated that self-defining memories (specific and integrated SDMs) may be predicted by neurocognition, associated with metacognition, the way one thinks about one’s abilities, and linked to goal outcomes. Neurocognition and metacognition are known predictors of poor functional outcome in psychosis, and recently metacognition was demonstrated to mediate between neurocognition, functional capacity, and functional outcome in first episode psychosis (FEP) (Davies, Fowler and Greenwood 2017). Self-defining memories may also have a role in predicting poor functional outcome. However, previous studies have only assessed those with chronic schizophrenia, none have looked at the relationship to functional outcome or pattern of SDMs in First Episode Psychosis. This study aimed to investigate the pattern of SDMs in FEP and the independent contribution of self-defining memories to outcome.

**Methods:**

This was a cross-sectional study involving a sample of 71 people with First Episode Psychosis who completed measures for neurocognition, metacognition (Metacognitive Assessment Interview and Beck’s Cognitive Insight Scale), self-defining memories, functional capacity (UCSD Performance-Based Skills Assessment) and functional outcome (hours spent in structured activity per week) using Time-Use Survey (Fowler et al., 2009). Research has demonstrated time spent in structured activity is 63.5 hours in healthy non-clinical population, 25.2 hours in a First Episode Psychosis sample, and 19.7 hours in a psychosis sample with delayed recovery (Hodgekins et al., 2015). Data was compared to a matched healthy control sample. It was hypothesised that self-defining memories would be less specific, less integrated and more negative in First Episode Psychosis compared to healthy controls, and self-defining memories would mediate between neurocognition and functional outcome in a multiple mediation model.

**Results:**

Self-defining memories reported by individuals with First Episode Psychosis were less specific, less integrated, and more negative, focused on relationships, failure and life threatening events, compared to matched healthy control group. Within the First Episode Psychosis sample, holding less specific memories was associated with engagement in significantly fewer hours of structured activity per week (14.9 hours for non-specific memories and 43.3 hours for specific memories), and this effect remained after controlling for neurocognition and metacognition. A multiple mediation model demonstrated that the specificity of SDMs mediated the relationship between neurocognition and functional outcome, independent of functional capacity and metacognition.

**Discussion:**

This study demonstrated that the types of self-defining memories reported are different between First Episode Psychosis and healthy controls, and may play a key role in functioning. This study was able to demonstrate a significant difference between the individuals with FEP reporting a specific compared to a non-specific memory on hours spent in structured activity. In such that participants who provided a specific memory were likely to have a better functional outcome and able utilise their neurocognitive ability to participate in more activities. Given these results, self-defining memories could be considered as a key factor to be explored within current FEP interventions.

